# A novel technique for protecting and cooling surrounding soft tissues when using bone cement

**DOI:** 10.1308/rcsann.2014.96.1.83a

**Published:** 2014-01

**Authors:** LM Pestana, D Hargreaves

**Affiliations:** University Hospital Southampton NHS Foundation Trust,UK

**Figure 1:**
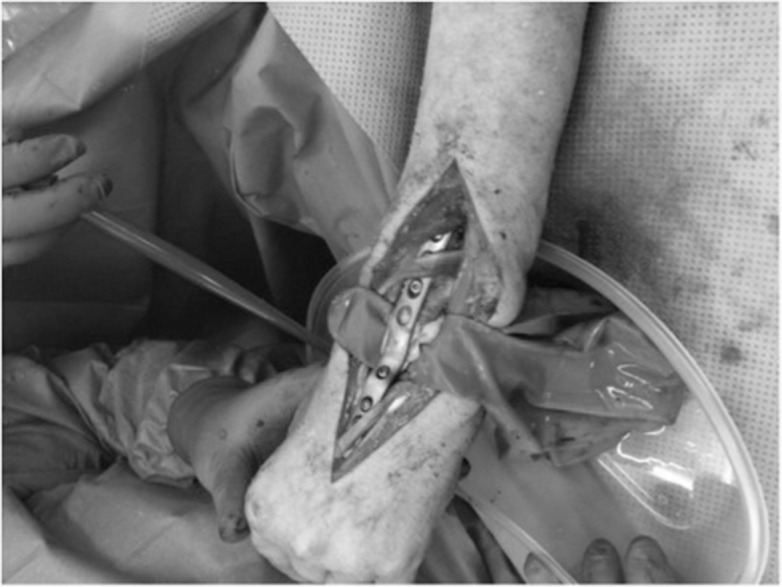
Insertion of partially water filled surgical glove to protect anterior wrist structures

**Figure 2:**
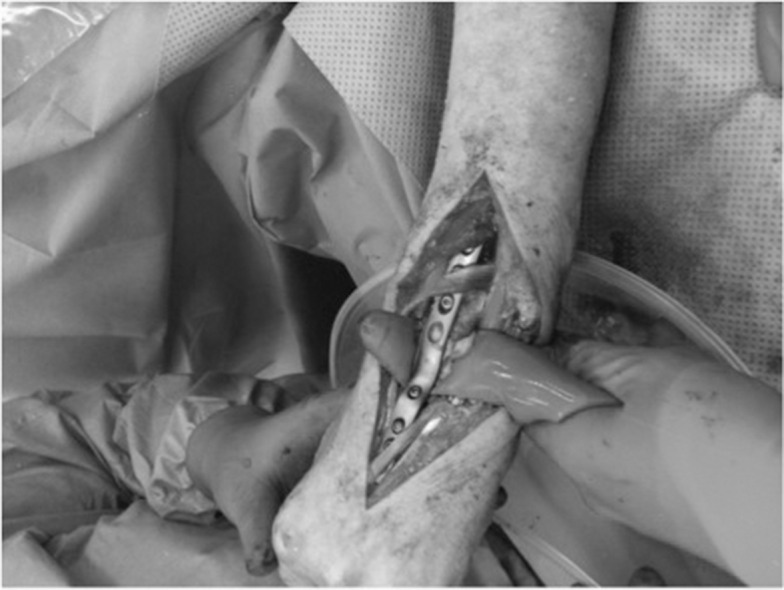
Squeezed glove

We describe a technique for protecting and cooling soft tissues while using cement in bony defects. Internal fixation using cement to augment neoplastic lesions is a recognised technique but the resulting exothermic reaction risks the surrounding tissues. We have used a surgical glove partially filled with water to protect volar structures of the wrist while cementing a distal radial lesion (Fig 1). Through intermittent squeezing, we additionally provided cooling (Fig 2). Other reported means of protecting from the setting cement are silicon sheets but these lack the cooling effect of our technique. This technique is applicable to other sites.

